# Triplets

**Published:** 1858-09

**Authors:** 


Triplets.—The following communication was sent to the Journal by Dr. J. Levergood, of Lancaster, Penna.:—

In the February and June numbers for 1856, of *The Medical Examiner*, the writer recorded the histories of two cases of triplet births, and also stated that, within the brief period of forty-eight months, Wrightsville, a town containing a population of less than fourteen hundred souls, had furnished three cases of the kind. Through the kindness of the attending physician, I am placed in possession of all the circumstances attending *another accouchement* of triplets in the above-named village, making the *fourth* in five years. I very much question whether the annals of obstetric medicine can furnish a parallel to this; indeed, it may as well be conceded at once, that, for "their generous fecundity," the ladies of Wrightsville stand pre-eminent and unrivaled. Having been requested to write a brief account of the last case, I

shall do so, without any further prefatory remarks.

Dr. Geo. K. Thompson was called, on the 23d of June, 1858, to attend Mrs. C., aged about twenty-eight, in labor with her third child. The labor progressed favorably, head presenting, and, in a short time, a living female child was born. There being evidences of a second fœtus in utero, the membranes were ruptured—the presentation being that of the nates—and, in due course of time, a still-born female infant was born. It appearing from examination per vaginam that there yet remained another child to be cared for, the bag of waters was ruptured, the vertex ascertained to be the presenting part, and, in three hours subsequent to the birth of the first, a third still-born female child was delivered. The third stage of parturition was completed by the extraction of a single placenta. The recovery was speedy and perfect.

These cases were equally distributed among the medical practitioners of the town, the first occurring in the practice of Dr. B. C. Floyd, the second in the practice of the writer—who formerly resided in Wrightsville—and the third and fourth cases, in the practice of Drs. J. A. and G. K. Thompson. Of the twelve children, there were four males and eight females; and of the whole number there are but three living.

